# Emotion through Locomotion: Gender Impact

**DOI:** 10.1371/journal.pone.0081716

**Published:** 2013-11-22

**Authors:** Samuel Krüger, Alexander N. Sokolov, Paul Enck, Ingeborg Krägeloh-Mann, Marina A. Pavlova

**Affiliations:** 1 Department of Pediatric Neurology and Developmental Medicine, Children's Hospital, Medical School, Eberhard Karls University of Tübingen, Tübingen, Germany; 2 Department of Psychosomatic Medicine and Psychotherapy, Medical School, Eberhard Karls University of Tübingen, Tübingen, Germany; 3 Center for Pediatric Clinical Studies, Children's Hospital, Medical School, Eberhard Karls University of Tübingen, Tübingen, Germany; 4 Werner Reichardt Center for Integrative Neuroscience, Eberhard Karls University of Tübingen, Tübingen, Germany; 5 Institute for Women's Health Baden-Württemberg, Eberhard Karls University of Tübingen, Tübingen, Germany; University of Muenster, Germany

## Abstract

Body language reading is of significance for daily life social cognition and successful social interaction, and constitutes a core component of social competence. Yet it is unclear whether our ability for body language reading is gender specific. In the present work, female and male observers had to visually recognize emotions through point-light human locomotion performed by female and male actors with different emotional expressions. For subtle emotional expressions only, males surpass females in recognition accuracy and readiness to respond to happy walking portrayed by female actors, whereas females exhibit a tendency to be better in recognition of hostile angry locomotion expressed by male actors. In contrast to widespread beliefs about female superiority in social cognition, the findings suggest that gender effects in recognition of emotions from human locomotion are modulated by emotional content of actions and opposite actor gender. In a nutshell, the study makes a further step in elucidation of gender impact on body language reading and on neurodevelopmental and psychiatric deficits in visual social cognition.

## Introduction

Every single day we are watching strangers passing by. We automatically determine not only speed, trajectory, and direction of their locomotion in order to avoid collisions and safely get through a crowd, but also spontaneously judge mood, intentions, dispositions and personality traits of walkers, which may be useful for a potential social interaction. Adult perceivers discern emotions and dispositions of others conveyed by point-light displays that reduce other kinds of information except for body motion [1]–[7]. Yet this ability seems to require a period of maturation during childhood [7]. In one of the initial studies in the field [8], youthful point-light gaits were reported to appear as more powerful and happier. Later a few attempts had been made to identify body motion parameters that are associated with the perceived social and personality traits [9]–[12]. Visual sensitivity to camouflaged point-light locomotion is modulated by the emotional content of gait with the highest sensitivity to angry locomotion [13], and the ability to recognize anger in displays portraying masked human locomotion is related to gait detection [14]. Moreover, in agreement with the assumption that biological motion processing serves a hallmark of social cognition [15], in typically developing adults and individuals with autistic disorders, the ability to reveal emotions from point-light body motion may be related to more basic capability for discrimination between canonical and scrambled biological motion [16], [17].

Emotional gender stereotyping appears to affect decoding of biological motion displays. Point-light displays depicting angry throwing a ball are often judged to be performed by men, whereas displays depicting sad throwing are referred to portray women [18]. Yet it is unclear whether the ability for veridical body language reading is impacted by gender. According to popular beliefs about female superiority in social cognition, there are some indications for sex impact on biological motion processing in non-human primates, common marmosets (*Callithrix jacchus*): females only exhibit curiosity to point-light biological motion displays [19]. Newly hatched female chicks are reported to exhibit a stronger preference for point-light biological motion of a walking hen (even over a walking cat) than their male peers [20]. This preference presumably reflects stronger affiliate tendencies in females. Gender congruency between perceivers and actors affects visual priming of camouflaged point-light locomotion [21], whereas alterations in biological motion processing with age appears to be unaffected by observers' gender [22]. Functional magnetic resonance imaging, fMRI, reveals enhanced brain activation during point-light biological motion processing in adult females as compared to males over the regions involved in social cognition (such as the temporal pole and amygdala) [23]. These sex differences are reported to be less pronounced in school-age youth.

Females excel in body language reading through expressive full-light (neck to knees or ankles) body motion video clips [24]. The first study on reading of point-light body language made use of displays representing knocking at a door with different emotional expressions [25]. In a three-alternative-forced choice paradigm, healthy female and male adults indicated whether a display portrayed happy, neutral, or angry knocking. The outcome shows that gender effects are modulated by emotional content of actions: Males excel in recognition accuracy of happy actions, whereas females tend to excel in recognition of hostile angry knocking and are substantially better in recognition of neutral knocking. Another study shows that females are more accurate in recognition of point-light activities (walking, jumping on the spot, kicking a ball, drinking from a bottle, and wiping the table), and tend to be faster in differentiation of canonical point-light biological motion from scrambled displays [16]. Most important, females are reported to surpass in some aspects of body language reading: they are faster in making judgments on whether point-light displays are happier, sadder, angrier, or not different from an emotionally neutral prime portraying the same activity, in other words, in discrimination of emotional from neutral body motion. The lack of gender impact on emotion discrimination accuracy may have been at least partly explained by a rather high performance level in both females and males. It appears plausible that gender effects are more evident in recognition of subtle rather than explicit, full-blown or exaggerated emotional expressions. For example, gender effects are reported to be more pronounced in recognition of facial emotional expressions of lower intensity [26] or in briefly exposed displays [27].

**Figure 1:**
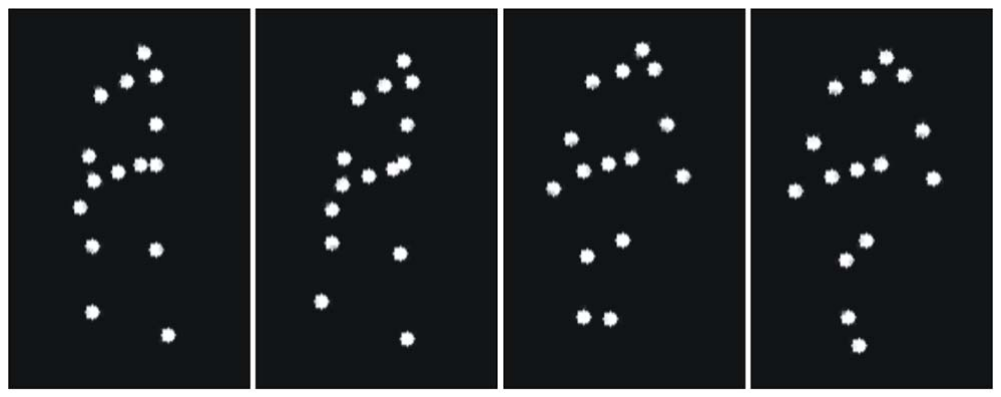
Illustration of stimuli. Four static images illustrating angry human walking as a set of dots placed on the main joints and head of an invisible actor body. Each display consists of 15 white dots presented against a black background. During locomotion, a walker was seen facing right in intermediate position (45°) between the frontal and sagittal view.

The present work intends to make a further step in clarification of whether gender affects body language reading by studying recognition of emotional human locomotion. More specifically, we ask (i) whether gender of observers affects recognition of emotions represented by human gaits; (ii) whether gender effects depend on emotional content of gait; and (iii) whether gender effects in recognition of human locomotion are impacted by actor gender. With this purpose in mind, healthy female and male adult observers were presented with point-light displays portraying human locomotion with different emotional expressions. We used a point-light methodology that helps to isolate information revealed by motion from other cues (shape, color, etc.). Perceivers saw only a few bright dots placed on the main joints of an invisible actor (Figure 1), so that all other clues except for motion characteristics were eliminated.

## Materials and Methods

### Participants

Fifty three adults, students of the University of Tübingen were enrolled in the study. Age of females (27 participants) was 23.15±1.1 years (median; 95% confidence interval), and of males (26 participants) was 24±1.33 years. There was no age difference between female and male participants (Mann-Whitney test, U = 407.5, p = 0.32). All observers had normal or corrected-to-normal vision. None had head injuries or medication for anxiety or depression, and a history of neurological or psychiatric disorders including autistic spectrum disorders and schizophrenia. They were run individually. None had previous experience with such displays and tasks. The study was conducted in line with the Declaration of Helsinki and was approved by the local Ethics Committee at the University of Tübingen Medical School. Informed written consent was obtained from all participants. Participation was voluntary, and the data were processed anonymously.

### Stimuli and procedure

Participants were presented with point-light displays portraying human locomotion. Display creation is described in detail elsewhere [28]. The displays were built up by using the Motion Capture Library. In brief, recording was performed using a 3D position measurement system at a rate of 60 Hz (Optotrak, Northern Digital Inc., Waterloo, ON, Canada). The matrix data for each frame was processed with MATLAB (The Mathworks Inc., Natick, MA, USA) into a video sequence. Each display consisted of 15 white dots visible against a black background (Figure 1). The dots were placed on the shoulder, elbow, and wrist of each arm; on the hip, knee and ankle of each leg; and on the head, neck, and pelvis of a human body. Each video consisted of 101 frames, and was presented at a rate of 60 frames per second. Each gait cycle was accomplished in 67 frames. As we supposed more pronounced gender effects would occur in recognition of subtle emotional expressions, we used brief stimulus duration. Each movie lasted for 1.68 s that corresponded to 1.5 walking cycle. During locomotion, a walker was seen facing right in intermediate position of 45° between the frontal and sagittal view. We used this intermediate trajectory of locomotion, because the sagittal view is often considered neutral in respect to possible social interactions, and the frontal view is reported to elicit ambiguous (facing backward or toward an observer) and often gender-dependent impressions of locomotion direction [29]–[32]. The walking figure was pelvis fixed to the middle of the screen.

Four females and four males served as actors. They were asked to walk with different emotional expressions (happy, angry, or neutral). All sets of stimuli were created from the same actors for avoiding variability in emotion portrayal. We chose to use animations with neutral, happy and angry motion primarily to enable comparison of the findings with the previous study on body language reading with a point-light knocking motion [25]. By using the Presentation software (Neurobehavioral Systems Inc., Albany, CA, USA), each video was displayed four times per experimental session resulting in 32 trials per emotion. The whole experimental session consisted of a set of 96 displays that were presented in a random order. A white fixation cross was displayed in the center of the screen for 3.32 s of inter-stimulus interval. Each session took about 10–15 min per participant. We used a three-alternative-forced choice paradigm. On each trial, participants indicated (by pressing one of three respective keys) whether the display portrayed happy, neutral or angry locomotion. No immediate feedback was given regarding performance.

## Results

Individual rates of correct responses (proportion correct) were submitted to a 2×3×2 repeated-measures analysis of variance, ANOVA (as assessed by the Shapiro-Wilk test, the data were normally distributed) with factors Gender of observers (female/male), Emotional expression (happy/neutral/angry), and Gender of actors (female/male). The outcome revealed that main effects of Gender of observers (F(1,51) = 0.01, ns) and Gender of actors (F(1,51) = 0.13, ns.) were non-significant, whereas a main effect of Emotional expression (*F*(2,102) = 60.28, *p*<0.0001) and interaction between the factors Emotional expression×Gender of actors (*F*(2,102) = 3.43, *p*<0.036) were significant. Post hoc analysis of simple effects revealed, however, that anger was not better recognized from displays portraying male as compared to female actors, and happiness from displays portraying female as compared to male actors. No difference was found in recognizability of neutral locomotion from movies portraying female and male actors. All other interactions were non-significant (Gender of observers×Emotional expression (*F*(2,102) = 1.18), Gender of observers×Gender of actors (*F*(1,51) = 1.13), Gender of observers×Emotional expression×Gender of actors (*F*(2,102) = 0.77)).

**Figure 2:**
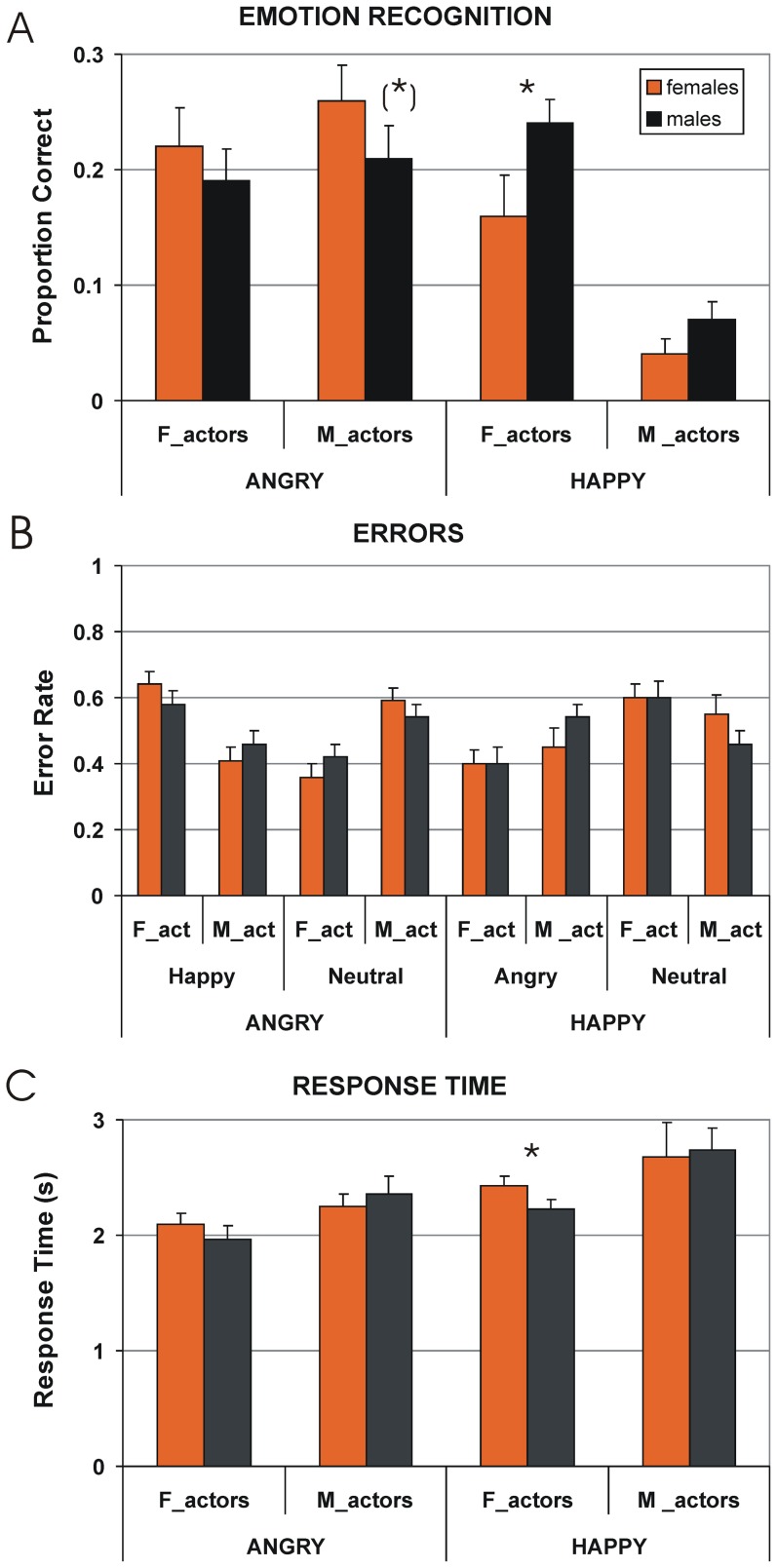
Recognition of subtle expressions of angry and happy point-light locomotion. **A**) Proportion correct: Males over-perform females in recognition accuracy of happy walking portrayed by female actors, whereas females exhibit a tendency to be better in recognition of angry locomotion expressed by male actors. **B**) Error rate: The lack of gender differences in error rate indicates that gender differences are not caused by gender-related bias for mistaking one emotion for another. **C**) Response time: Males are faster than females in responding to happy walking portrayed by female actors. Asterisks indicate significant gender differences, whereas asterisks in brackets indicate a tendency. Vertical bars represent ±SE.

As we expected more distinct gender impact on recognition of subtle emotional expressions (see Introduction), we set 41% cut-off (determined as a mean value of display recognizability) for recognition of emotions through human locomotion, and focused on analysis of the displays that were recognized below this level. No gender effects were found in recognition accuracy of displays that were recognized above the cut-off. All neutral displays were recognized above the cut-off, and, therefore, our analysis was limited to displays depicting angry and happy locomotion. Proportion of correct responses in emotion recognition is represented in Figure 2A. As can be seen, males surpass females in recognition of happy walking portrayed by female actors (*U* = 475.1, *p*<0.016), whereas females exhibit a tendency to be better in recognition of angry locomotion expressed by male actors (*t*(51) = 1.68, *p*<0.098). The data, therefore, reveal a lack of overall advantage of females in recognition of emotion through human locomotion.

To ensure that gender effects in emotion recognition were not due to gender-related bias for mistaking one emotion for another, we performed an error analysis. As seen in Figure 2B, in both females and males, happy locomotion expressed by female actors was primarily mistaken for neutral locomotion (mean error rate ± standard deviation, 0.6±0.04 and 0.6±0.05 for females and males, respectively; gender difference: *t*(51) = 0.03, *p* = 0.97). When happy locomotion expressed by female actors was misperceived as angry locomotion, a lack of gender differences was also found (0.4±0.04 and 0.4±0.05 for females and males, respectively; *t*(51) = 0.03, *p* = 0.97). In turn, when angry locomotion portrayed by male actors was erroneously recognized, both females and males primarily mistook it for neutral locomotion without gender differences (0.59±0.04 and 0.54±0.04 for females and males, respectively; *t*(51) = 0.97, *p* = 0.34). When angry locomotion performed by male actors was misperceived for happy gait, no gender differences were found in error rate (0.41±0.04 and 0.46±0.04 for females and males, respectively; *t*(51) = 0.97, *p* = 0.34). The lack of gender differences in error rates suggests that gender effects in recognition accuracy of emotions through locomotion found in the present study are not due to gender-related bias for misperceiving one emotion for another.

For response time analysis, a 2×2×2 repeated-measures ANOVA was performed on individual values (as assessed by the Shapiro-Wilk test, the data were normally distributed) with factors Gender of observers (female/male), Emotional expression (happy/angry), and Gender of actors (female/male). This analysis reveals a main effect of Emotional expression (*F*(1,51) = 182.39, *p*<0.0001). This outcome indicates that for both females and males, swiftness of response to emotional locomotion depends on its emotional content. As further seen from Figure 2C, the fastest response was given to angry locomotion represented by female actors, and the slowest response to happy locomotion represented by male actors. This suggests that for both female and male participants, displays representing subtle expression of a happy man were most difficult to recognize, whereas recognition of subtle angriness expressed by female actors was the easiest. A main effect of Gender of actor was significant (*F*(1,51) = 109.09, *p*<0.001). On overall, emotions portrayed by female actors were more readily recognizable than emotions expressed by male walkers. Males were not only better in recognition of happy walking portrayed by female actors, but also faster in responses to these displays than females (Figure 2C; *t*(51) = 2.42, *p*<0.019).

## Discussion

The outcome of the present study suggests that gender impacts recognition of subtle emotions from human locomotion only, and this occurs in a complex way. The gender effects in recognition of subtle emotions are modulated by the emotional content of locomotion and opposite actor gender: Males surpass females in recognition accuracy and readiness to respond to subtle expressions of happiness performed by female actors, whereas females exhibit a tendency to be better in recognition of angry locomotion expressed by male actors. The lack of gender differences in error rates indicates that gender effects in recognition accuracy are not caused by gender-related bias for mistaking one emotional expression for another. The findings agree with previous evidence on gender effects in recognition of emotions from point-light displays portraying knocking: Male observers over-perform in recognition of happy knocking, whereas females tend to better recognize hostile angry motion [25]. This earlier study however, did not address the issue of whether gender effects are associated with gender of actors. The present work suggests that gender effects in body language reading can be modulated not only by emotional content of body motion, but also by (opposite) actor gender.

The present data appears to challenge the recent theoretical reasoning suggesting that production of actions may be intimately linked with understanding of intentions and actions of others [33], [34]. From the mirror neuron system point of view, one would expect that observers would over-perform in recognition of emotions expressed by actors of the same gender (i.e., females would be better in recognition of emotions expressed by female actors, and males in recognition of emotions expressed by male actors), because they have common or more similar motor programs engaged in emotional expressions, and therefore can understand emotional locomotion of others “from the inside” [33, p.264]. Actually, such common motor programs may facilitate biological motion perception: gender congruency between an observer and a runner during the visual priming improves detection of direction of a point-light runner embedded into a complex simultaneous dynamical mask [21]. Motor expertise may enhance perception of point-light biological motion displays portraying dance [35]: female dance experts are better in similarity discrimination of point-light dance elements expressed by female actors (when observers and performers share not only visual experience, but also common motor program for dance performance) than male experts (when observers and performers share enriched visual experience only). Acquired motor skills in dance performance also specifically affect brain activity during action observation [36], [37]. However, facilitation effect of gender congruency was not observed in the present study dealing with some aspects of social cognition.

Although social cognition is presumably associated with active interactions and immediate reactions, and, therefore, body language reading is likely associated with motor programs, we did not observe facilitation effects of gender congruency in recognition of subtle emotions from locomotion. Our findings rather agree with assumptions based on the evolutionary or ecological accounts that imply gender-specific socio-cultural differences. Indeed, higher sensitivity of male observers even to subtle happiness expressed by female walkers might suggest perceptual significance of positive emotions in potential partner selection [38]. In addition, a considerable amount of research has documented that both female and male observers are especially tuned to anger expressions depicted in different kinds of point-light biological motion [1], [3], [13], [14], [25] and in faces and full-body displays [39], presumably because perceiving anger is of particular relevance for one's own well-being and helps to avoid critical situations. Bearing in mind that from the evolutionary and socio-cultural points of view, female roles are often associated with offspring care providing, it appears that women might be not only more sensitive to anger expressions in body language, but also exhibit higher sensitivity than males even to subtle clues of anger expressed by males because they may signal potential danger. Future research should confirm whether gender effects in body language reading persist with other repertoires of body movements, and with other arrays of emotions.

At first glance, the outcome of the present study appears to contradict the findings reported by Alaerts and colleagues [16] about female superiority in some aspects of body language reading in point-light displays: although females do not differ from male observers in accuracy, they tend to be faster in discrimination of emotional (happy, angry, sad or neutral) point-light body motion from neutral displays. This apparent discrepancy may be explained by methodological differences in the tasks (discrimination vs. forced choice paradigm), movies duration, and variety of portrayed point-light actions.

Future research should be directed at uncovering sex differences in brain activity during body language reading. First of all, it is unclear whether the neural circuits underlying body language reading are sex specific. The existing findings on sex differences in the social brain are either limited to investigation of static and dynamic faces (for recent review, see [40]) or extremely sparse. In males, greater fMRI brain activation over the extrastriate body area, superior temporal sulcus, fusiform gyrus, pre-supplementary motor area, and premotor cortex (with a lack of behavioral differences) is reported for a full-body male threatening versus neutral displays [41]. Brain activation during visual processing of point-light biological motion overlaps topographically, especially, in the right temporal cortex, with the network engaged in visual perception of agency and social attribution in Heider-and-Simmel-like movies representing motion of geometric shapes [42], [43]. Yet sex differences are not manifested in the neural circuitry underpinning visual processing of social interaction in Heider-and-Simmel-like animations. Gender impact is evident only in the regions engaged in perceptual decision making: the magnetoencephalographic (MEG) oscillatory induced gamma response over the left prefrontal cortex boosts later in males [43]. Furthermore, the time delay in peak MEG activation in males corresponds to longer response time to the Heider-and-Simmel animations as compared with control stimuli [43].

Growing neuroimaging evidence points to sexual dimorphism of the brain [44]–[46], also in the white matter underlying brain connectivity between different areas [47], [48]. Investigation of sex differences in body language reading would help to clarify the nature of neurodevelopmental and psychiatric disorders characterized by impairments in social cognition. Many of these disorders are gender-specific: females and males are differently affected in terms of prevalence and clinical picture. Males have a higher risk for developing autistic spectrum disorders than females, with a sex ratio of about 4:1 [49]. Neuroanatomy of autism is reported to differ between females and males [50]. For Down syndrome, the reported sex ratio is 1.28 [51], and for fragile X syndrome, the ratio is 2 [52]. Males are at a 14–20% higher risk for premature birth [53] and of its complications in the brain development and cognition [54]. On the other hand, depression is approximately twice as common in females as in males [55]. Females are more often affected by anxiety disorders with a ratio of 2∶1 or even 3∶1, and gender differences occur already in childhood increasing with age [56], [57]. Although in most of these disorders some aspects of biological motion processing and body language reading are reported to be impaired [58]–[62], gender impact on these impairments is largely unknown. Clarification of gender effects in body language reading and underlying brain networks would provide novel insights into understanding of gender vulnerability to psychiatric and neurodevelopmental deficits in social cognition [15].
